# The Physiological Importance of Glucosinolates on Plant Response to Abiotic Stress in *Brassica*

**DOI:** 10.3390/ijms140611607

**Published:** 2013-05-30

**Authors:** María del Carmen Martínez-Ballesta, Diego A. Moreno, Micaela Carvajal

**Affiliations:** 1 Plant Nutrition Department, Centre of Edaphology and Applied Biology of Segura (CEBAS-CSIC), Campus of Espinardo, Building 25, Murcia E-30100, Spain; E-Mail: mcarvaja@cebas.csic.es; 2 Phytochemistry Lab, Food Science and Technology Department, Centre of Edaphology and Applied Biology of Segura (CEBAS-CSIC), Campus of Espinardo, Building 25, Murcia E-30100, Spain; E-Mail: dmoreno@cebas.csic.es

**Keywords:** abiotic stress, glucosinolates, isothiocyanates, plant tolerance, secondary metabolism

## Abstract

Glucosinolates, a class of secondary metabolites, mainly found in Brassicaceae, are affected by the changing environment. This review is focusing on the physiological significance of glucosinolates and their hydrolysis products in the plant response to different abiotic stresses. Special attention is paid to the crosstalk between some of the physiological processes involved in stress response and glucosinolate metabolism, with the resulting connection between both pathways in which signaling mechanisms glucosinolate may act as signals themselves. The function of glucosinolates, further than in defense switching, is discussed in terms of alleviating pathogen attack under abiotic stress. The fact that the exogenous addition of glucosinolate hydrolysis products may alleviate certain stress conditions through its effect on specific proteins is described in light of the recent reports, but the molecular mechanisms involved in this response merit further research. Finally, the transient allocation and re-distribution of glucosinolates as a response to environmental changes is summarized.

## 1. Introduction

Glucosinolates, a class of secondary metabolites, are nitrogen- and sulfur-containing compounds mainly found in Capparales and almost exclusively in Brassicaceae, which include *Brassica* crops of economic and nutritional importance, as well as the model plant, *Arabidopsis thaliana* [1,2]. Glucosinolates are chemically stable under normal conditions, but when plant tissues and cells are damaged, they are hydrolysed by the enzyme myrosinase, resulting in several degradation products, including isothiocyanates, nitriles, thiocyanates, epithionitriles and oxazolidines [3]. Chemical factors, such as pH, availability of ferrous ions and the presence of myrosinase-interacting proteins, determine the final products [2,4]. Among these products, special attention have been paid to isothiocyanates, because they are involved in plant defense against pathogens and herbivores, whereas in humans, the consumption of vegetables containing glucosinolates, such as broccoli, kale and Brussels sprouts, may confer protection against cancer [5]. However, the physiological significance of glucosinolates and their degradation products in plants is not completely understood. Although the glucosinolate-myrosinase system is assumed to play a role in plant-herbivore and plant-pathogen interactions, several reports have considered glucosinolates as a sink for nutrients, like nitrogen and sulfur [6–8]. However, when *Brassica* plants were exposed to excess sulfur amounts, the sink capacity of glucosinolates was limited [9,10].

In numerous reports, the effect of abiotic stresses on the primary metabolic processes has been well documented (*i.e.*, photosynthesis, growth and metabolism of antioxidants) [11,12]. However, environmental factors also influence secondary metabolism (Table 1). Generally, when plants are stressed, secondary metabolism may increase, because growth is often limited more than photosynthesis, and carbon fixation is predominantly invested to secondary metabolites production [13]. However, although previous studies indicate that environmental factors, such as light [14], temperature [15] salinity [16,17] and drought [18,19], may modify glucosinolate composition, their physiological role in response to abiotic stress is not known.

It has been reported that suppression of aliphatic glucosinolates by RNA interference (RNAi) in *Arabidopsis* plants provoked changes in protein and metabolites involved in physiological processes, such as photosynthesis, oxidative stress and hormone metabolism [12]. These results reflected that pathways involved in physiological responses were closely connected to glucosinolate metabolism that can be also modifying by abiotic stress factors [12]. In addition, understanding chemical changes in plants upon abiotic stress and the ecological implications for the plant-herbivore interactions is needed.

In this review, the current status on the effect of different abiotic stresses on the production of glucosinolates is presented and discussed in the context of the recent results of their involvement in the abiotic-biotic stress interaction, as well as the plant response to environmental changes when glucosinolates are exogenously added. In addition, an update of the glucosinolate transport and allocation upon abiotic stress is reported.

## 2. Changes in the Glucosinolate Profile under Abiotic Stress: Implications for Plant Adaptation

### 2.1. Salinity

Salinity can be considered as the major abiotic stress affecting plant physiology and, thus, plant development [20,21]. One of the effects of salinity is the alteration of the secondary metabolism involving signal molecules, oxidative stress and intermediary reactions. The secondary metabolites, glucosinolates, have been shown to increase in plants when salinity stress is present above the tolerance levels [16,22–24]. However, the different physiological stage of the plant or the level of tolerance to salinity and the individual glucosinolate response (in relation to glucosinolate-myrosinase system) needs a detailed analysis to discuss the response of glucosinolates to salt stress.

Therefore, even though previous works evidenced that salinity (40 mM) highly increased the total glucosinolate content in broccoli inflorescences [22], these authors also observed that when the levels of salinity were high (80 mM), the increase in glucosinolates was reduced. These results could be related to the strong metabolism alteration focused in turgor adjusting and leading to a high growth reduction [17,25]. The fact that glucosinolates were accumulated under low water potential when the leaves have to maintain turgor suggested that during salinity stress, the primary metabolism and growth were restricted, but not the secondary metabolism and the production of glucosinolates. It was suggested that the increase in glucosinolates was related to the synthesis of osmoprotective compounds.

The variation in the amount and pattern of glucosinolates into the plant has been related to developmental stage [26]. As a response to salinity, the increase of total glucosinolates was shown to be more pronounced in the florets than in the young fully expanded leaves, probably due to a higher *de novo* synthesis or the increased transport to this physiological sink via the phloem [17]. The lack of an active metabolism for glucosinolate synthesis in the old leaves to a sink organ may explain the glucosinolate levels after the salt treatments. In another study, it was shown that a divergent composition of glucosinolates exists in different organs of the halophyte, *Thellungiella*, at different developmental stages of its lifecycle as a response to salinity [27], which reinforces the idea that glucosinolates could be synthesized *de novo* during the processes of growth.

Glucosinolates degradation and turnover is carried out by the activity of myrosinases [28,29]. Tissue damage brings myrosinase (cytoplasm) in active contact with glucosinolates (vacuole) [30,31]. However, the complexity of the regulation of the glucosinolate-myrosinase system has been pointed out, since no relationship has been observed between the glucosinolate level as a result of altered myrosinase activity in salinity stressed plants [24]. In this study, an increase in glucosinolates degradation was expected to be observed as a consequence of membrane damage by salt stress indicated by high relative electrolyte leakage. Therefore, the lack of relation between myrosinase activity and glucosinolate levels supported the hypothesis that salinity results in an alteration of metabolic activity producing an increase in glucosinolate content.

It has been also reported that salinity stress response may show lifelong latency in the way in which a plant allocates resources to growth, defense and reproduction. Therefore, as salinity stress affects the priorities of the plant in all the stages of its lifecycle, changes in glucosinolates synthesis as a result of cellular signaling could be observed [32]. However, the fact that glucosinolates act as signals themselves can also be considered.

Therefore, the mechanism of glucosinolate turnover regulation under salinity still is not completely clarified. The effect of salinity on biosynthesis and metabolism on individual glucosinolate merits further investigations. Furthermore, the function of glucosinolates under a salinity stress situation, further than in defense switching, in terms of alleviating the pathogens attack, deserves more attention.

### 2.2. Drought

Water stress increased the glucosinolate accumulation in *Brassica* species—*Nasturtium officinale* L. [18]; *Brassica oleracea* L. var. *capitata* [19]; *Brassica oleracea* L. var. *italica* [33,34]; *Brassica napus* L. [35]; *Brassica rapa* ssp. rapifera L. [36]; and *Brassica carinata* L. [37]—in agreement with the prediction of “protein competition model”, where drought is expected to reduce some vegetative growth parameters with the subsequent increase of secondary metabolites at the expense of primary metabolism [38].

Although the induction of glucosinolates accumulation by drought conditions has been reported as part of the plant response to stress through the process of osmotic adjustment [37], contradictory results have been observed in the literature when high drought (30% of the amount of water received by well-watered plants) had no effect on the concentration of total glucosinolates in *Brassica oleracea* L. var. *gemmifera* (Brussels sprouts) [39] or in *Brassica napus* L. under mild drought stress [35], whereas water deprivation produced significant glucosinolate reductions in *Brassica oleracea* [40–42] and in the rosette leaves of *Arabidopsis thaliana* L. [43]. Therefore, the intensity and duration of drought appear to be an important factor in the accumulation of each specific glucosinolate, as well as the developmental stage of the plant when the stress is applied.

On the other hand, it is known that plant-pathogens or plant-herbivores interactions that affect glucosinolates content may be influenced by stressful environments modifying the mechanisms of plant defense [44,45]. Therefore, several studies have been focused on the interaction between drought stress and herbivore damage to elucidate the involvement of phytochemicals in this response and its ecological implications. In *Brassica oleracea*, Gutbrodt *et al.* [39] showed that two lepidopteran herbivores preferred drought-stressed plants to the well-watered, although glucosinolate concentrations did not change. These authors suggested that other compounds involved in the drought stress-response may regulate feeding preferences. Khan *et al.* [46] studied the interaction of different water regimes and two aphid pest species (*Brevicoryne brassicae* and *Myzus persicae*) in broccoli. The plant response with enhanced glucosinolate content after feeding by *Myzus persicae* depended on plant water availability, whereas the plant response to *Brevicoryne brassicae*, with increasing glucosinolate levels, was independent of the water status. Similar results were found in *Arabidopsis* plants [47], where *M. persicae* damage reduced the accumulation of glucosinolates in most water treatments, with the downregulation of the jasmonic acid (JA) pathway.

In Brussels sprouts, glucosinolate levels increased 62% under the combination of drought stress and root herbivory and were positively correlated with the aphid development time, which probably varied, due to the altered relative water content (turgor pressure) of the plant under drought [48]. However, the effect of environmental conditions on herbivore-plant interactions was dependent on the level of stress, and the response to stress intensity is not always linear.

In recent reviews, the importance of the abiotic-biotic stress interaction has been highlighted [49–51]. In the response to water stress, abscisic acid (ABA) plays a definitive role promoting adaptations, such as stomatal closure. The antagonistic effect of ABA, JA and ethylene (ET) on defense response against plant-pathogens is well known. Thus, the inhibition of disease resistance induced by ABA would be the reason of pathogen preferences for water-stressed plants. However, recent results showed that the expression of biotic stress markers that are involved in indole glucosinolate biosynthesis were significantly higher in the leaves of well-watered *Arabidopsis* plants than in plants grown under mild water deficit (40% reduced soil water content), whereas no significant differences were found in the expression levels of genes involved in hormone and signaling response to drought stress, such as ABA and ET, under both set of conditions [52]. These results pointed to the complexity of the response to the abiotic and biotic stress interaction.

Also, it has been proposed that abiotic stress may increase the delivery of glucosinolates from the vacuole to the cytosol in leaf cells of *Arabidopsis thaliana* or enhance the activity of myrosinase or its substrate affinity in such a way that the hydrolysis products of the glucosinolates (their cognate isothiocyanates) could lead to the inhibition of inward K^+^ channels in the guard cells to avoid water loss by stomatal closure [53]. According to this, a recent report showed that the application of exogenous allyl-isothiocyanate to the leaves of *Arabidopsis thaliana* induced stomatal closure, leading to the suppression of water loss and preventing the possibility of fungal invasion [54].

Beattie [50] reported some of the genes affected by the crosstalk of both abiotic and biotic signaling pathways, indicating some of the proteins that have been identified as possible components of both pathways. This author underlined how the control of water movement could be a mechanism for pathogen restriction. In this sense, the involvement of the membrane water channels or aquaporins is suggested.

Furthermore, recently, CML42, a calmodulin-like protein, has been identified as a protein that coordinates both herbivory and abiotic stress responses in *Arabidopsis* [55]. In the proposed model for the roles of CML42, it was suggested that the protein acts as a negative regulator of plant defense against *Spodoptera littoralis* by ABA accumulation under drought stress and increased aliphatic glucosinolate levels among others signaling mechanisms [55].

Finally, Siemens *et al.* [56,57] suggested that glucosinolates may condition the processes that determine regional range boundaries for *Boechera stricta*, contributing to the restricted spatial distribution typical of most species of mustard. The authors proposed that the drought stress tolerance associated with range boundaries may compromise the defense evolution, and both pathways, ABA response and JA/ET signaling, together with the glucosinolate regulation are involved in the tradeoff and the development of species range limits.

### 2.3. Extreme Temperatures and Light Cycling

Glucosinolates content varies in response to temperature and light quality [14]. The seasonal variation for the glucosinolate content in different *Brassica* sp., such as radish [58,59], oilseed rape [60,61], turnip [36,62] and cabbage [63–67] has been reported. In these studies, it was observed that spring season conditions, such as moderate temperatures, low humidity, high light intensity and longer photoperiods, induced higher glucosinolate accumulation than autumn/winter season conditions. Thus, elevated temperatures have been shown to increase glucosinolate levels in *Brassica rapa* [68], and a positive relationship between soil temperature and glucosinolates has also been documented in *Brassica oleracea* [63,65]. However, Justen *et al.* [69] observed some discrepancies with the results presented by Charron and Sams [65], referring these differences to distinct genotypes and growth environments. Thus, an interaction between temperature, solar radiation or plastic mulch properties may condition glucosinolate content in greenhouse or field experiments. Also, it must be considered that, in general, indole glucosinolates are more sensitive to elevated temperatures than aliphatic or aromatic glucosinolates [3,70]. Therefore, the contribution of each individual and specific glucosinolate to the variation of total glucosinolate levels by the temperature regime results in decisive importance.

Rosa *et al.* [71] observed that glucosinolate exhibited circadian rhythms varying during 24 h. This variation was more evident when the temperature was optimum for growth and development. However, at higher temperatures, glucosinolate changes were due to the effect of temperature rather than to the photoperiod. Thus, it has been evidenced that glucosinolate variation throughout the day follows similar patterns to other plant components in response to external factors. However, the results observed in *Brassica oleracea* where the glucosinolate levels decreased during the day and increased during the night [72,73] were contradictory with those found in *Arabidopsis* where an enhanced accumulation of glucosinolates during the day was found in relation to the glucosinolate content in the night [74]. Differences were attributed by the authors to the distinct developmental stage of the plants, as well as the main herbivores associated with both species, depending on their preferences for feeding during the day or by contrast if they were nocturnal insects.

Also, Pérez-Balibrea *et al.* [75] demonstrated that broccoli sprouts growing under light conditions had higher total glucosinolates content than those grown in darkness, but different results were found concerning the effect of light intensity and quality on glucosinolate concentrations [76] depending on plant genotype, as well as the particular glucosinolate.

In several reports have been determined the transcription factors involved in the glucosinolate modulation by the temperature and the cycling light. Recently, in *Brassica rapa*, some MYB transcription factors have been identified as responsible for glucosinolate accumulation under elevated temperature [68]. Similar regulation of the aliphatic glucosinolate transcripts levels by the light was observed in plants with modulated expression of MYB factors [77–79]. However, the aliphatic and indolic MYB factors have been shown to be regulated differentially in *Arabidopsis* plants by the light cycling [74], and thus, the transcription factor HY5 acts as a repressor of the aliphatic MYB factors and as an activator of indolic MYB factors, being at the same time involved in the sulfate assimilation. This fact pointed out the co-regulated coordination of glucosinolates and sulfur metabolism by the cycling light [74]. The interconnectivity between metabolism and the circadian rhythm to optimize carbon allocation has been suggested in a recent review [80], where it has been pointed out that the gene, *AOP2*, codifies a biosynthetic glucosinolate enzyme that controls glucosinolate conversion into another, altering circadian clock regulation [81]. In addition, discrepancies between the glucosinolates content and the expression level of the genes involved in their synthesis during the photoperiod [82,83] indicated that the regulation of glucosinolates biosynthesis was not only due to gene expression, but also, the glucosinolate turnover exerted influence.

It has been shown that an *Arabidopsis* mutant deficient in glucosinolate metabolism presented reduced levels of the heat-shock stress protein, Hsp90, and less tolerance to elevated temperatures [84]. Recently, it has been demonstrated that the exogenous addition of two isothiocyanates, phenethyl- and allyl-isothiocyanates, enhanced heat tolerance in *Arabidopsis* plants by increasing the expression level of heat shock proteins and H_2_O_2_ accumulation [85,86]. It was determined that the mode of action of phenethyl-isothiocyanate (PEITC) was similar to salicylic acid (SA), a heat tolerance inducer that inhibits the catalase activity in plants and produces the H_2_O_2_ accumulation [87], but the molecular mechanisms behind the recognition of both compounds may be different and worth study.

Therefore, glucosinolates and their hydrolysis products, the isothiocyanates, may induce heat tolerance by modulating the plant physiological status in a similar way to stress-acclimating processes. An additional function of glucosinolates and isothiocyanates in the physiological responses of plants to high temperatures could be proposed, in addition, to the well-known defense function against pathogens.

### 2.4. Nutritional Deficiencies

Identification of standard nutritional requirements of *Brassica* crops is particularly important, due to the impact of providing added-value plants products with superior health-promoting benefits. As glucosinolates are nitrogen (N)- and sulfur (S)-containing plant secondary metabolites, S and N supply and the right balance between N:S have a clear effect on their concentration in *Brassica* plants.

Brassicaceae requires relatively large amounts of N for optimum growth and production of high quality inflorescences [88]. The amount of N found to give optimal yield varies greatly, due to soil properties, climatic conditions and product requirements [89]. However, excessive or low amounts of N supply may cause some physiological disorders, like hollow stem, and some pathological problems that may alter glucosinolates concentration [90]. Excess N usually produces a decrease in total glucosinolates [15]. However, opposite of what can be thought, some studies showed that broccoli plants grown with an insufficient N supply showed an increase in total glucosinolates if S fertilization is not limiting [91]. This has been discussed by the high coordination of both N and S assimilatory pathways; the deficiency of one element represses the other pathway [92]. When S is limited, *O*-acetyl serine can accumulate, whereas if N is limited, the increase of *O*-acetyl serine is inhibited. The effect of both *O*-acetyl serine and reduced S compounds have been shown to act antagonistically [93]. Therefore, low S supply combined with optimal N fertilization could thus lead to accumulation of *O*-acetyl serine and reduced cysteine synthesis, resulting in a lack of precursors for glucosinolate synthesis.

Also, different responses have been described according to the specific glucosinolate. N fertilization had a significant impact on the content of aliphatic or indolic glucosinolates in *Eruca sativa* L. [94]. While aliphatic glucosinolates responded negatively to N fertilization, indole glucosinolates showed a positive response. However, in other studies, high rates of N (120 or 240 kg/ha) application resulted in an increase of the aliphatic glucoraphanin and the indolics, glucobrassicin and neoglucobrassicin [90]. These differences have been discussed in terms of the effects of N on biomass production and its consequences for glucosinolates biosynthesis.

An increased S supply has been shown to result in higher levels of total glucosinolates in *Brassica rapa* [95] and of individual glucosinolates, such as glucoraphanin and glucoraphasatin [96], sinigrin, glucobrassicanapin, gluconapin and progoitrin in *Brassica juncea* L. [97]. Interestingly, other works showed no significant differences in total glucosinolates, aliphatic or indolic glucosinolates in different cultivars and breeding lines between poor (15 kg/ha) and very high (150 kg/ha) S fertilization when the rest of conditions were optimal [98]. These results highlighted the relevance of N stress *versus* S stress in relation to glucosinolates metabolism and revealed that S nutritional stress leads to glucosinolate synthesis alteration.

Potassium (K^+^) deficiency has been shown to increase oxylipins and glucosinolates levels in *Arabidopsis* plants [99]. These authors showed higher levels of glucosinolates in the roots compared to the shoots, the root glucosinolates being little affected by K deficiency. A differential function for glucosinolates in the roots and shoots was proposed, as well as a restricted role of jasmonic acid (JA)-signaling in the shoot, mediating glucosinolate synthesis after the K deficiency. Similarities with the induced JA pathway by herbivores may suggest the role of glucosinolates to enhance plant defense ability under K starvation. However, in another report, an early induced K deficiency reduced plant growth and reproduction, as well as decreased the glucosinolates content in the seeds of *Brassica rapa* [32]. The contradictory results showed the plasticity of plant response to sustained or short-term K deficiency and should be taken into account for further investigation on glucosinolate production and enrichment.

The effect of micronutrient stress has been related to glucosinolates synthesis. In this sense, selenium (Se) fertilization has been related with S nutrition in the study of its effect on the synthesis of glucosinolates [40,100]. Kim *et al.* [100] observed a high accumulation of Se in all varieties with increased glucosinolates concentration in only some of the studied cultivars via MeJA-pathway mediated indolic glucosinolate accumulation in broccoli. Also, in former papers, boron deficiency has been related to the increase of indolic glucosinolates [101], suggesting that the increase of the flux through the oxidative pentose phosphate and shikimic acid pathways led from tryptophan to accumulated glucosinolates.

## 3. Glucosinolate Organ or Tissue Allocation and Transport under Abiotic Stress

Plant resistance to abiotic and biotic stress involves an optimized allocation of resources, as well as direct protection of plant tissues by secondary metabolites [102–104]. In this way, glucosinolates are usually present in all parts of the plant, but concentration is already variable, depending on the different plant tissues and developmental stages under normal situations [105,106]. Generally, the highest levels of glucosinolates were found in young leaves and reproductive tissues, such as siliques and seeds, whereas the glucosinolate content declined in mature leaves [105]. However, in a recent review based on the determination of glucosinolate content in the roots and shoots of 29 plant species, it has been documented that roots have higher concentrations and diversity of glucosinolates than shoots [107]. Whereas the biotic factors that influence glucosinolate allocation have been widely studied [108–110], less is known concerning the distribution and transport of glucosinolates under abiotic stress. In fact, there are only a few studies concerning the relation between the variation of shoot and root glucosinolate profiles and concentration and the different environments where plants grow.

Abiotic stressors, such as salinity, drought, nutrient deficiency and acidity, may change defense allocation patterns in *Brassica rapa* [32]. Thus, the concentration of gluconapin, was significantly higher in leaves and seeds of acidity-treated plants and significantly lower in seeds of nutrient-deficient plants. The results were in agreement with previous reports, where the early response of glucosinolates to nutrient deficiency was studied in different tissues [111]. Therefore, the lower availability of nutrients, especially N and S as glucosinolate precursors, may alter glucosinolate tissue allocation. Thus, plants may catabolize their glucosinolates and use the released sulfur to assist primary metabolism, such as protein synthesis in a specific organ. In *Arabidopsis*, this loss of glucosinolates from old leaves has been attributed to catabolism or transport to other organs in response to sulfur demand [26].

In aerial parts, the glucosinolate concentrations varied with different factors, including temperature, time of day, water content and nutrient supply [72,73,112,113]. Under controlled conditions, the glucosinolate concentrations in the leaves of *Brassica* species oscillated when the plants were sown at different periods of time during several months, attributing these differences to abiotic seasonal changes [113].

Soil properties, such as pH, may modify glucosinolate concentrations in the leaves of kale [15,114,115], pointing to a distinct distribution of each glucosinolate in the root or the shoot tissues.

A direct allocation cost was assigned to glucosinolate production in *Arabidopsis*, with an increase of 15% in photosynthetic energy, to produce a set of 30 glucosinolates [116]. This fact may justify the transient allocation and re-distribution of glucosinolates, suggesting a temporal and spatial transport of glucosinolates as the response to environmental changes [116]. Therefore, as plants are unable to escape unfavorable environments, allocation of glucosinolates could be one strategy to cope with stressful conditions with relative low energy cost. However, the biological function of a particular glucosinolate in specific tissues requires more investigation.

## 4. Concluding Remarks

**Figure 1:**
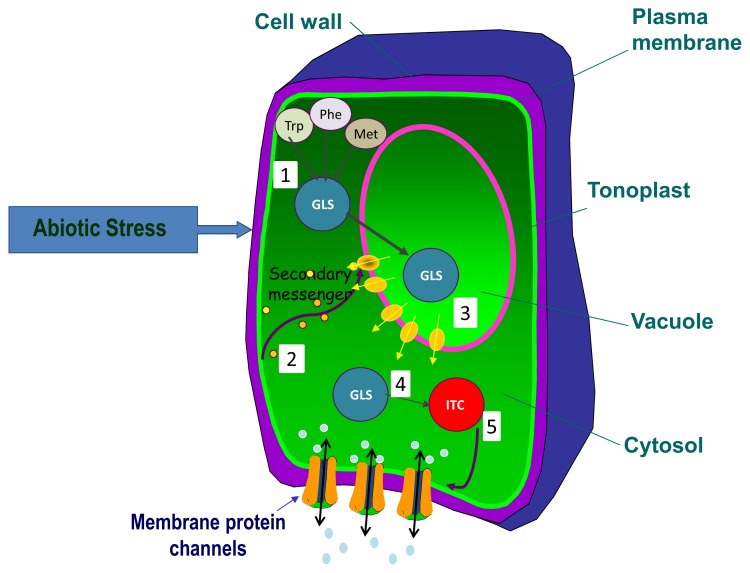
Effect of abiotic stress on glucosinolates (GLS)/allyl-isothiocyanates (ITC) content. (1) The chain-elongation steps of glucosinolates biosynthesis are affected by abiotic stress; (2) Stress perception may lead to increased synthesis of any secondary messenger in the cytosol, facilitating the release of the glucosinolates to the cytoplasm from the vacuole (3). The activity of plant myrosinases will product (allyl-) isothiocyanates (4) that might regulate the transduction or accumulation of other transporters or channels in the plasma membrane (5).

As conclusive remarks, abiotic stresses, such as salinity, drought, extreme temperatures, light and nutrient deprivation, alter the glucosinolate profiles of plants through different mechanisms (Figure 1), where distinct signaling molecules may be involved. The tight relationship between some physiological processes under abiotic stress and the glucosinolate metabolism suggest that these secondary metabolites may have auxiliary roles associated to these physiological events. This involves a clear connection between the pathways, and glucosinolates may participate on the signaling mechanisms. The fact that glucosinolate follows diurnal variations similar to other plant molecules in response to environmental and external factors may support this idea.

The intensity and duration of the abiotic stress, as well as the developmental stage of the plant at the moment of the imposed stress, are important factors in the accumulation of each specific glucosinolate. This fact conditions the subsequent plant-pathogen interactions, and the plant water availability is decisive in influencing herbivore feeding or the attack by pathogens.

The addition of exogenous glucosinolate hydrolysis products (isothiocyanates) under drought or elevated temperatures alleviated the adverse effects of these unfavorable environments, but the molecular mechanisms involved merit research in order to be clarified. Finally, the fact that a transient allocation and re-distribution of glucosinolates in response to environmental changes is observed could give an indication that glucosinolate-specific function under abiotic stress is still unclear and requires further attention.

## Figures and Tables

**Table 1:** Effect of different abiotic stress conditions on glucosinolate production of different Brassicaceae.

Abiotic stress conditions	Plant cultivar	Glucosinolate content	References
Saline stress			
NaCl (40, 80 mM), during two weeks	*Brassica oleracea* L. var. *italica*	Increase	López-Berenguer *et al.*, 2008 [22]
NaCl (20,40, 60 mM), during 5 d	*Brassica rapa* L.	Increase	Steinbrenner *et al.*, 2012 [32]

Drought			
Severe stress two weeks	*Brassica oleracea* L. var. *capitata*	Increase	Radovich *et al.*, 2005 [19]
Severe stress two weeks	*Brassica oleracea* L. var. *italica*	Increase	Champolivier and Merrien 1996 [33]
Severe stress more than one week	*Brassica napus* L.	Increase	Jensen *et al.*, 1996 [35]
Mild stress-25% of available water	*Brassica rapa* ssp. rapifera L.	Increase	Zhang *et al*., 2008 [36]
Mild and severe stress (40, 23, 17 and 15% of available water)	*Brassica carinata* L.	Increase /No effect	Schreiner *et al.*, 2009 [37]
Mild stress (30% of available water)	*Brassica oleracea* L. var. *gemmifera*	No effect	Gutbrodt *et al.*, 2012 [39]

Mild stress	*Brassica napus* L.	No effect	Jensen *et al.*, 1996 [35]
Mild and severe stress (40%–45% of available water)	*Brassica oleracea* L.	Decrease	Gutbrodt *et al.*, 2011a Khan *et al.*, 2011a [41,42]
Severe stress	*Arabidopsis thaliana* L.	Decrease	Ren *et al.*, 2009 [43]

Temperature			
Elevated temperature (21–34 °C)	*Brassica rapa* L.	Increase	Justen and Fritz 2013 [68]
Low-medium temperature (15–27 °C)	*Brassica rapa* L.	Decrease	Justen and Fritz 2013 [68]
Elevated temperature (32 °C)	*Brassica oleracea* L.	Increase	Charron *et al.*, 2004, 2005 [63,65]

Light cycling			
14 h/10 h d/n *	*Brassica oleracea* L.	Decrease during day/increase during night	Rosa *et al.*, 1997, 1998 [71,73]
16 h/8 h d/n or continuous darkness	*Arabidopsis thaliana* L.	Increase upon light /decrease upon darkness	Huseby *et al.*, 2013 [74]
16 h/8 h d/n or continuous darkness	*Brassica oleracea* L. var. *italica*	Increase upon light	Pérez-Balibrea *et al.*, 2008 [75]

Nutrient availability			
N-limitation (1 gr N pot^−1^)	*Brassica oleracea* L. var. *italica*	Increase	Schonhof *et al.*, 2007 [91]
S-supply (60 kg S ha^−1^)	*Brassica rapa* ssp. *rapifera* L	Increase	Li *et al.*, 2007 [95]
S-supply (150 kg/ha)	*Brassica oleracea* L. var. *italica*	No effect	Vallejo *et al.*, 2003 [98]
S-limitation (15 kg/ha)	*Brassica oleracea* L. var. *italica*	No effect	Vallejo *et al.*, 2003 [98]
K-deficiency( lack KNO_3_ for two weeks)	*Arabidopsis thaliana* L.	Increase	Troufflard *et al.*, 2010 [99]
K-deficiency ( lack of nutrient solution for five days)	*Brassica rapa* L.	Decrease	Steinbrenner *et al.*, 2012 [32]
Se-supply (5.2 mM Na_2_SeO_4_)	*Brassica oleracea* L. var. *italica*	Increase	Kim *et al.*, 2011 [100]
B-deficiency (9–12 μg gr DW^−1^)	*Brassica oleracea* L. var. *italica*		Shelp *et al.*, 1992 [101]

* d/n: day/night rhythm.
